# Statistical determination of service quality gaps in primary health care in Guayas, Ecuador

**DOI:** 10.1371/journal.pone.0299994

**Published:** 2024-03-15

**Authors:** Miguel A. Bustamante U., Michelle Tello, Mauricio Carvache-Franco, Orly Carvache-Franco, Wilmer Carvache-Franco

**Affiliations:** 1 Facultad de Economía y Negocios, Escuela de Ingeniería Comercial, Universidad de Talca, Dos Norte 685, Talca, Chile; 2 Sistema de Posgrado, Universidad Católica de Santiago de Guayaquil, Av. Carlos Julio Arosemena Km. 1½ vía Daule, Guayaquil, Ecuador; 3 Universidad Bolivariana del Ecuador, Durán, Ecuador; 4 Universidad Espíritu Santo, Km. 2.5 Vía a Samborondón, Samborondón, Ecuador; 5 Facultad de Ciencias Sociales y Humanísticas, Escuela Superior Politécnica del Litoral, ESPOL, Campus Gustavo Galindo Km 30.5 Vía Perimetral, P.O. Box 09-01-5863, Guayaquil, Ecuador; Universidad San Francisco de Quito, ECUADOR

## Abstract

The present work determines the gaps between expectations and perceptions about the quality of the service that patients and families receive in primary health care (PHC) in Guayas, Ecuador. A descriptive, cross-sectional and non-experimental study was carried out, primarily prospective with respect to expectations and retrospective with respect to perceptions of service quality. For its development, a random sample of 533 users from the northern and southern urban sectors of the city of Guayaquil was determined, who were asked to answer a questionnaire. Their responses were collected using a seven-point scale intended to determine magnitudes of gaps, which were confirmed using the Wilcoxon test. The results reveal a significant gap between women, specifically those over 21 years of age who have studied at the technological and university level, and those who work. In general, the five dimensions of quality present significant gaps, highlighting that the lowest gap occurs when the medical professional listens attentively and treats the patient with kindness. Instead, the largest gap occurs because there are no available or easily accessible times for medical appointments. Finally, the dimension that indicates the gaps that service providers best resolve are the empathy items, recording the smallest deviations; On the contrary, the reliability dimension presents the greatest deviation, thus showing higher degrees of dissatisfaction, in both cases significant.

## 1. Introduction

Primary Health Care (PHC) is the first level of contact with users who require quality services [1, 2], particularly care [3, 4] as well as forecasting and monitoring of the morbid population [2, 5], they value the quality of the service when they perceive it [6, 7] and appreciate the concrete [4, 8] more than the intangible [9–11], regardless of whether the benefits are hospital or PHC level [8, 12, 13], which are especially valued in times of pandemic as was Covid-19 [14].

Exemplifying cases of service quality perception, in Spain we have sought to increase patient satisfaction with personalized services [15] and in Turkey we have sought to improve user perception through technologies [16], however, dissatisfaction gaps persist [17, 18].

The gap is expressed by contrasting expectations and perceptions on the part of patients or family members [3, 19] regarding the characteristics of a product—service [3, 7] as those identified in a multidimensional model [20] that determined it through attributes and factors [1, 10] capable of reporting satisfaction [4, 13] as dissatisfaction [10, 21] defining identifiable gaps, consequently, this work seeks to determine the level of expectations in contrast to the levels of perceived satisfaction with the services provided at the PHC level [5, 6, 22], on the one hand, identifying relevant items and then significant factors [8, 13, 19] that allow us to collect, with the greatest possible certainty, the gaps between expectations and perceptions [3, 23].

The construct in its original version contained 97 items grouped into ten dimensions. Subsequently, through factor analysis, it was synthesized into 22 items grouped into five dimensions that measure the expected and perceived quality of service [6, 20], providing the scientific community with an instrument that has been widely used. used in the health sector in South American countries such as Brazil [3, 24], Chile [25], Colombia [26] and Mexico [27]. In turn, applications were identified in Malaysia and Pakistan in Asia [19], in addition to studies carried out in China [28] and India [29], among others, confirming the effectiveness of the construct.

Finally, some studies of gaps between expectations and perceptions were found in Argentina [22], in Brazil [17] and in Mexico [18], evidencing the effectiveness of gap studies [4, 21]. Consequently, and based on the concepts analyzed and the evidence of application of the Servqual construct, this work assumes the objective of determining the gaps between expectations and perceptions regarding the five service quality factors of users of PHC services. in the context of Guayas, Ecuador.

## 2. Methodology

The Project was ethically approved by the Santiago de Guayaquil Catholic University of Ecuador. Informed consent was given in writing as part of the questionnaire.

This non-experimental, exploratory and quantitative study seeks to determine the gaps between expectations and perceptions of the interviewees [20, 28]. As an inclusion criterion, patients or family members of legal age were included, randomly chosen to adequately reflect the diversity of the population and who receive benefits at the PHC level [4, 21].

### 2.1. Sampling and instrument

The sampling procedure adopted in this study was probabilistic in nature, following a confidence level of 95%, a maximum variance of 50% and a tolerable margin of error of 5%, in accordance with the guidelines outlined by Lloret et al. [30] and Hernández-Sampieri and Mendoza [31]. Given the nature of the research focused on perceptions, the criterion of number of people per item (N/p) was applied with proportions of 10:1 and 5:1 to determine the suitability of the sample [30]. In addition, the extension of the instrument was considered, which consists of 22 items to evaluate expectations and another 22 to collect perceptions, according to the recommendations of Valencia-Arias et al. [8] and Tang [32]. Consequently, when applying the N/p criterion, it was stipulated that the sample had to be substantial (n ≥ 440), thus guaranteeing the reliability, power and statistical significance necessary for the gap analysis, in accordance with the guidelines of Juárez et al. [33].

The Servqual instrument [20] used in the present study was previously subjected to a translation and adaptation process to the health context, according to the methodology proposed by Valencia-Arias et al. [8]. This process considered the specificity of the primary health care (PHC) environment, as conceptualized by Macarayan et al. [2]. Likewise, the application of the construct in the Chilean context by Bustamante et al. [25], who, by verifying the sequentiality between expectations and perceptions in the field of health, confirmed the effectiveness of the items and the explanatory capacity of the factors from the perspective of patients and family members, corroborating previous findings by Papanikolaou and Zygiaris [1].

However, in order to ensure accuracy in data collection, the instrument was subjected to an exhaustive review by a panel of experts composed of academics and health professionals, both from the public and private spheres. This review process aimed to adapt the items to the linguistic nuances of users seeking care in the field of primary health in the Guayas region, Ecuador. This linguistic adaptation approach follows the recommendations of Greenslade and Jimmieson [34] and Tripathi and Siddiqui [23].

As a result of these considerations, a questionnaire structured in three parts was obtained. The first scale evaluates patients’ expectations regarding the quality of services, while the second scale measures their perceptions, using in both cases a seven-point scale (1:7), where 1 indicates total disagreement and 7 represents total agreement, in accordance with the indications of Lloret et al. [30] and Hernández-Sampieri and Mendoza [31]. In general, scales above 7 points allow us to assume that the data behave normally as ratified by Freiberg et al. [35] and ratified by Pruzan [36]. Finally, the third part of the questionnaire collects demographic information about the sample., thus contributing to an adequate characterization of it.

The dimensions of the Servqual instrument, according to the proposal of Parasuraman et al. [20], cover the factors a) tangible elements, which refer to physical facilities, equipment and appearance of personnel; b) reliability, associated with the ability to perform the promised service reliably and accurately; c) responsiveness, related to the willingness to help customers and provide prompt service; d) security, which encompasses the competence, knowledge and courtesy of employees, as well as their ability to instill trust, credibility and security; and, finally, e) empathy, referring to the critical components of affectivity, closeness and understanding of providers towards users [13, 28].

These five dimensions of service quality are detailed in 22 critical items specific to health services, which have previously been studied using factor analysis techniques [25], determining that three expectations factors and three perception factors explain 63.6% and 61.97% of the total variance, respectively, providing a strategic guide for administrative decisions in the field of health services. In this context, the present study is positioned as an additional and profound contribution to the analysis of the quality of the health service at the level of primary health care (PHC), with the objective of determining with greater specificity the existing gaps between the items of expectations and perceptions through the application of the Wilcoxon test, as will be discussed later [33, 37].

### 2.2. Data collection, analysis, and reliability

Data collection was carried out over a period of three months in various health centers and medical clinics located in Guayaquil, Ecuador. The execution of the field work involved formal coordination with the authorities of each health care center through a written communication addressed to the directors. In this correspondence, the relevant information was provided and the essential documentation of the project was delivered along with its objectives, following the guidelines established by [32].

The interviewers, in accordance with the guidelines of Hernández-Sampieri and Mendoza [31], contacted the participants in the respective care centers, randomly choosing patients or family members. During this process, a contact procedure was implemented that included obtaining informed consent from the interviewees. Their responses were collected while ensuring the anonymity and confidentiality of the information, which was recorded in a condensed manner in an anonymous database, in accordance with the recommendations of Valencia-Arias et al. [8]. The questionnaire, designed to evaluate perceptions and expectations related to the quality of health services, was administered in an estimated time period that ranged between 25 and 30 minutes, thus guaranteeing efficient and exhaustive data collection.

The collected data was subjected to analysis using SPSS v.22 software, enabling the overview of the sample demographics and the accuracy of the service quality gap by calculating perceptions minus expectations (P-E), according to the precepts of Freiberg et al. [35] and Lloret et al. [30]. The statistical evaluation of the results was carried out by applying a frequency test for two independent sets of expectations and perceptions, considering both as integral parts of the same population, in accordance with the methodologies proposed by Shankar and Singh [37]. and Makowski et al. [38].

Next, the contrast of the results was carried out using the Wilcoxon [39] test (W) to compare the related and paired samples of expectations and perceptions in relation to the quality of hospital service. This test allowed us to determine if there were significant differences or gaps between both measurements. To do this, the differences between pairs of the 22 perceptions minus expectations items were calculated. The results in absolute value were ordered from lowest to highest, adding the ranges of positive and negative differences separately. The W test was applied by adding the lower of the two values of the sum of the ranks (positive or negative). The comparison of this value with the table of critical values made it possible to accept or reject the null hypothesis (H0). The null hypothesis establishes the absence of a significant difference between the two paired samples, while the alternative hypothesis (H1) indicates the presence of a significant difference, following the guidelines of Gibbons and Chakraborti [40].

Divergences between pairs of data evaluate perceptions in relation to expectations, as indicated by Hernández-Sampieri and Mendoza [31]. Furthermore, given the non-parametric nature of the tool used, it is pertinent to contrast the average range of the items and their corresponding factors in the related samples, with the purpose of verifying the presence or absence of significant differences that may be due to chance, in accordance with the recommendations of Shankar and Singh [37]. The use of the W index turns out to be effective for comparing pairs of data from populations that do not necessarily follow a normal distribution, as addressed in the contributions of Makowski et al. [38]. Likewise, it is necessary to point out that the records were ordered from smallest to largest, according to the Likert scale used in this study (1:7), according to the guidelines of Juárez et al. [33].

In a last step, with the objective of verifying the consistency and reliability of the instrument, Cronbach’s Alpha coefficient was applied [30]. This analysis was carried out individually for each of the five dimensions of the construct, as well as for the 22 items of the instrument. The established acceptance threshold was high, being equal to or greater than 0.70, in accordance with the criteria proposed by Freiberg et al. [35], Juárez et al. [33], and Makowski [38]. The choice of this threshold reflects the search for high reliability in the measurements obtained through the instrument used in the research.

## 3. Results

First, a synthesis of the descriptive statistical analysis is presented, and then the contrasting of expectations and perceptions of the construct that measures the quality of service is presented [20].

### 3.1. Description of the sample and reliability of the dimensions of expectations and perceptions

Based on the minimum sample criterion that was determined ex—ante following the proportion (N/p), it reached 440 cases, however, for the development of the research it was decided to exceed this minimum by obtaining 582 questionnaires, which were reviewed. to check for inconsistencies and completeness of records by using the frequency function in SPSS [32, 38]. In this way, the sample was refined, confirming 533 valid cases, which allow us to exceed the estimated minimum sample.

In this sample context, the demographics determined that 77.3% are women, confirming the fact that it is women who accompany patients and are the ones who assume family care tasks [41, 42] of both minors and their respective partners at the end of life [43] precisely when they go to request health services. The ages by range of the total sample are between 21 and 45 years, recording that 44.7% declare the marital status of common law. Regarding education, 53.7% claim to have achieved intermediate to higher level education.

### 3.2. Reliability analysis of quality of service factors

In general, the reliability and consistency evaluations concerning the five dimensions of the construct, both in relation to expectations and perceptions, reveal considerable statistical significance, exceeding the established threshold of 0.70. In general terms, the overall reliability of the instrument is notable, with a Cronbach’s alpha coefficient of 0.96 for the 22 items related to expectations and a value of 0.94 for perceptions. Likewise, the reliability of the five dimensions, both in the domain of expectations and perceptions, shows high levels of consistency. As an example, an alpha coefficient of 0.86 is identified for the Reliability dimension (Fia) and 0.89 for Security (Seg) among the expectations. Regarding perceptions, an alpha coefficient of 0.78 is recorded for the Tangible Resources factor (Rt), and the results of 0.88 for Security (Seg) corroborate that this last dimension exhibits significant levels of consistency, as presented in the Table 1.

**Table 1:** Reliability of the scale and service quality factors.

Reliability of Scales/Subscales	α Cronbach	Number of items	Scales/Subscales	α Cronbach	Number of items
Expectations	0.96	22	**Percepciones**	**0.94**	**22**
Reliability	0.86	5	Reliability	0.84	5
Security	0.89	4	Security	0.88	4
tangible resources	0.88	4	tangible resources	0.78	4
Response capacity	0.87	4	Response capacity	0.83	4
Empathy	0.87	5	Empathy	0.86	5

### 3.3. Gap analysis of service quality factors

When examining the mean scores and standard deviations obtained for the five factors presented in Table 2, it is evident that the values assigned to each dimension exhibit different behaviors on a scale of 1 to 7 points.

**Table 2:** Comparison of average expectations and perceptions.

Dimensions	Average expectations ± std	Average perceptions ± std	Gap P-E
Reliability (Fia)	6.47 ± 0.64	6.82± 0.42	-0.36± 0.65
1. The appointment with the medical professional was carried out at a specific time and date	6.66 ± 0.80	6.30 ± 0.85	-0.36
2. The medical professional explained the care procedures in an orderly and clear manner	6.75 ± 0.64	6.48 ± 0.74	-0.27
3. A good service was given since the first time you came	6.79 ± 0.53	6.49 ± 0.82	-0.30
4. The doctor or nurse introduces the diagnostic procedures	6.83 ± 0.50	6.60 ± 0.74	-0.23
5. There are times available and easy to get for the doctor’s appointment	6.88 ± 0.42	6.48 ± 0.94	-0.40
**Security (Seg)**	**6.72 ± 0.54**	**6.90 ± 0.32**	**-0.18 ± 0.53**
6. The medical professional inspires confidence	6.83 ± 0.53	6.70 ± 0.63	-0.13
7. You feel safe with the treatment provided by the medical professional	6.87 ± 0.42	6.73 ± 0.62	-0.28
8. The medical professional is kind	6.90 ± 0.38	6.74 ± 0.65	-0.17
9. The medical professional confidently responds to your questions and concerns	6.91 ± 0.33	6.75 ± 0.59	-0.16
**Tangible Resources (Rt)**	**6.72 ± 0.47**	**6.89 ± 0.36**	**-0.17 ± 0.48**
10. Necessary, modern, and optimal equipment	6.90 ± 0.38	6.51 ± 0.80	-0.38
11. Consultation room is clean and comfortable	6.87 ± 0.44	6.77 ± 0.56	-0.10
12. The medical professional has an appropriate personal presentation	6.89 ± 0.40	6.83 ± 0.45	-0.06
13. The consultation room has the necessary materials for diagnosis	6.91 ± 0.36	6.78 ± 0.55	-0.14
**Response capacity (Cr)**	**6.76 ± 0.51**	**6.90 ± 0.35**	**-0.14± 0.53**
14. The medical professional informs you exactly about your admission and next check-up	6.89 ± 0.37	6.78 ± 0.53	-0.12
15. The medical professional attends you at the appropriate time	6.89 ± 0.39	6.65 ± 0.84	-0.24
16. The medical professional is always willing to help you	6.89 ± 0.42	6.81 ± 0.54	-0.08
17. The medical professional is never too busy to answer your questions	6.91 ± 0.39	6.79 ± 0.54	-0.12
**Empathy (Emp)**	**6.79 ± 0.47**	**6.90± 0.31**	**-0.11 ± 0.46**
18. The medical consultation room offers individualized attention	6.88 ± 0.45	6.73 ± 0.70	-0.15
19. The medical consultation room has convenient working hours	6.90 ± 0.39	6.77 ± 0.58	-0.13
20. The medical professional listened carefully and treated you kindly	6.89 ± 0.40	6.82 ± 0.53	-0.07
21. The medical professional showed interest in solving your health problem	6.92 ± 0.36	6.83 ± 0.50	-0.08
22. The medical professional clearly understands what you need	6.89 ± 0.39	6.81 ± 0.62	-0.09
**Total quality**	**6.69 ± 0.45**	**6.89 ± 0.28**	**-0.20 ± 0.43**

Additionally, it is evident that the Security factor (Seg) exhibits an average of 6.72 points in expectations, contrasted with a value of 6.90 in perceptions. This disparity results in a gap of -0.18 points, indicating the presence of unmet expectations among users of the health system. Similarly, the Tangible Resources (Rt) factor presents expectations with an average of 6.72 points, in contrast to perceptions that reflect an average value of 6.89, thus generating a gap of -0.17 points. This finding suggests the need to improve the quality of certain aspects within this factor to reduce the disparity between expectations and perceptions. Finally, the Responsiveness factor (Cr) presents an average of expectations of 6.76 points, compared to a level of perceptions that reaches an average of 6.90 points. This gap results in a gap of -0.14 points, highlighting the importance of properly managing the variables that make up this dimension.

### 3.4. Gap analysis of service quality variables

**Fig 1:**
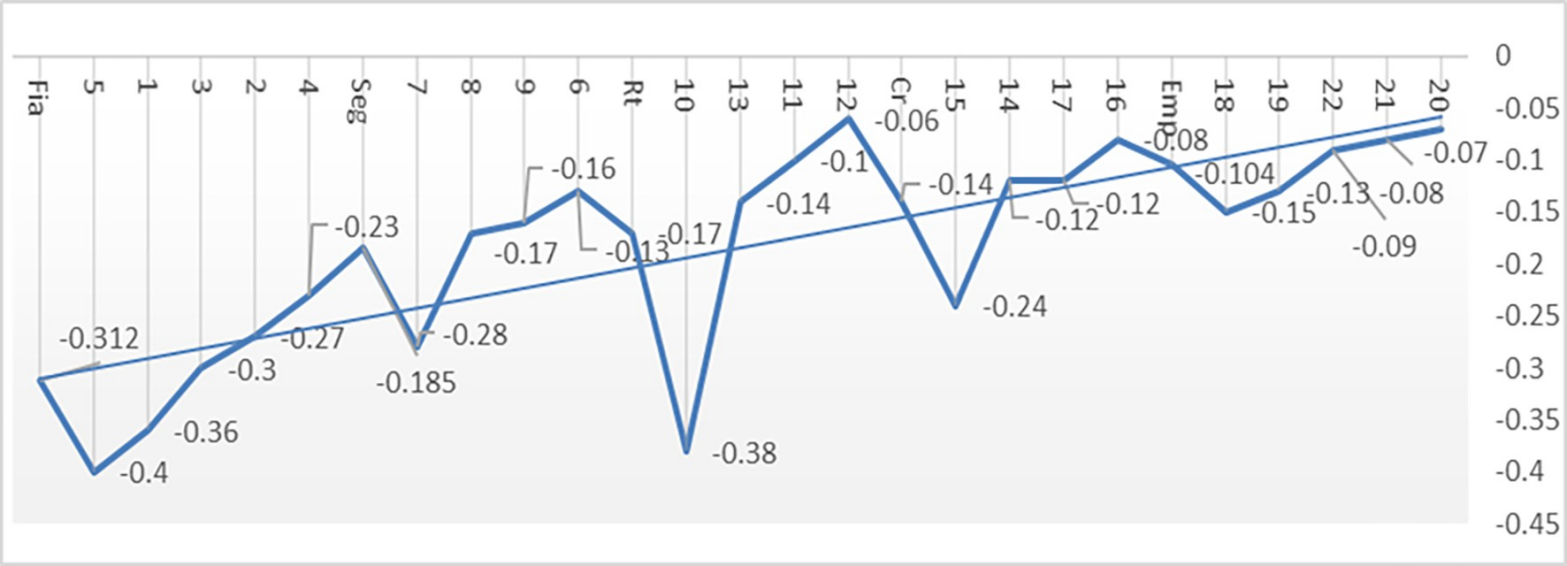
Gaps of the perceptions items regarding expectations.

When examining the disparities present in the 22 items of the instrument (Table 2), it is noted that all evaluations exhibit negative gaps, varying between -0.40 for item 5, "availability and accessibility of hours for medical consultation", and -0.07 for item 1, "the medical consultation offers individualized care", as illustrated in Fig 1. The most modest expectations were recorded in the reliability dimension, evidenced, for example, in the variable related to "perform consultation at the stipulated time and date" (6.66 ± 0.80), as well as in the perception of whether the clinical provider "explains the care procedures or not" (6.75 ± 0.64), and in relationship with the attitude of providers when "providing the service for the first time to users" (6.79 ± 0.53). In contrast, the highest expectation is manifested in the dimension of empathy, especially regarding the doctor’s interest (or lack of interest) in addressing patients’ problems (6.92 ± 0.36).

As shown in Table 2, certain variables in the instrument exhibit intermediate positions. In the Safety factor (Seg), the item that evaluates the perception that the patient or family member "feels safe with the treatment provided by the medical professional" reflects notable expectations (6.87 ± 0.42) and similar perceptions (6.73 ± 0.62). The resulting average gap (-0.28) indicates a relative demand for intervention by managers. In the Tangible Resources (Rt) dimension, the variable that addresses the relevance of the media, specifically, "necessary, modern and optimal equipment", raises expectations almost to the maximum (6.90 ± 0.38), while perceptions indicate a challenge (6.51 ± 0.80). Addressing this relatively demanding gap (-0.38) involves allocating resources to expensive applications, particularly in the healthcare field, where such resources are inherently scarce in institutions, both public and private. On the other hand, with regard to the Responsiveness factor (Cr), the variable related to the timely assistance of the medical professional presents significant levels of expectations (6.89 ± 0.39) and close perceptions (6.65 ± 0.84), giving rise to a gap of medium magnitude and relative demand (-0.24), especially if it is sought to be addressed through logistics management.

### 3.5. Trend analysis of service quality gaps

In general terms, Fig 1 illustrates the gaps of the items (1 to 22) and dimensions (Fia, Seg, Rt, Cr, Emp), represented by a straight line that progressively decreases from considerable gaps in the reliability factor (Fia = 5, 1, 3, 2, 4), indicative of lower levels of service quality and higher degrees of dissatisfaction, until reaching reduced gaps in the empathy factor (Emp = 18, 19, 22, 21, 20). The latter denote a greater proximity between expectations and perceptions of service quality, with values close to zero, although still marginally insufficient, indicating low magnitudes of dissatisfaction. For example and in greater detail, the most significant discrepancies were observed in the dimensions of Reliability (Fia), especially in relation to the consultation with the medical professional at a specific time and date (0.36), as well as in the difficulty of availability and relative ease of obtaining medical appointments (-0.40). Similarly, in the Tangible Resources dimension (Rt = 10, 13, 11, 12), more notable deviations were identified in relation to the necessary, modern and optimal medical equipment observed in care (-0.38). Likewise, the item linked to the care of the medical professional who assists (or not) at the corresponding moment (-0.24), belonging to the Responsiveness dimension (Cr = 15, 14, 17, 16), also showed significant deviations.

In contrast, smaller gaps are evident in the empathy factor (Emp), given that perceptions exhibit a notable proximity to expectations. This phenomenon is illustrated, for example, in item 20, which addresses whether the medical professional listened attentively and treated the patient kindly (-0.09), in item 18, referring to the convenience of the medical consultation time (- 0.08), and finally in item 18, linked to the option of individualized care offered (or not) by the medical consultation (-0.07). This convergence between expectations and perceptions is attributed to the high value assigned by patients and their families to the proximity, respect and affectionate care that health service providers in the field of Primary Health Care (PHC) ultimately provide. instance.

In general terms, the gaps identified in Fig 1 offer crucial indicators for the management of service quality in Primary Health Care (PHC), and are of high importance, given that users experience a varied range of disparities between their expectations and resulting perceptions. Particularly, the specific action of booking appointments deteriorates the promptness of care and decreases the anticipated quality of service. Likewise, users also identify a smaller gap in relation to the tangibility of the medical consultation, specifically in relation to the space allocated to personalized care offered by PHC service providers to patients or their families, which is perceived below expectations.

### 3.6. Demographic analysis of expectations and perceptions

As detailed in Table 3, expectations and perceptions exhibited slightly higher values in the female gender, although without reaching statistical significance (0.79). In contrast, in the case of the male gender, slightly wider but statistically significant gaps were observed (p). In relation to the age groups, no significant gaps were evident in the segment of those under 20 years of age (0.50), but they were in the other age ranges (21–25; 26–30; 31–45; 46–60).; and over 61 years old). Likewise, in terms of schooling, significant gaps were identified with a significance level of 5% (p) among those who indicated primary and secondary studies, while among those who declared technological and university studies, the gaps were significant with a level of 1% significance (p). Finally, the evaluation of gaps in service quality did not show significance among people who do not work (0.48), but significant gaps were detected among those who are employed.

**Table 3:** Gaps between expectations and perceptions according to demographics and dimensions of service quality.

Variables	Expectations	Perceptions	Gap P—E	value p (W)
Gender				
Male	6.78	6.58	-0.21	0.79
Female	6.91	6.72	-0.19	***
Age				
Less than 20 years old	6.90	6.68	-0.21	0.50
21 to 25 years old	6.89	6.63	-0.25	***
26 to 30 years old	6.91	6.73	-0.18	***
31 to 45 years old	6.86	6.71	-0.15	***
46 to 60 years old	6.85	6.66	-0.20	***
More than 61 years old	6.81	6.64	-0.36	***
Schooling level				
Elementary school / High school	6.93	6.71	-0.21	0.04
Technological / University	6.64	6.52	-0.12	***
Employment situation				
Works	6.92	6.71	-0.23	0.48
Does not work	6.87	6.69	-0.18	***
Gaps between expectations and perceptions by dimensions of service quality				
Dimension	Perception	Expectations	Gap	value p (W)
Reliability (Fia)	6.47 ± 0.64	6.82± 0.42	-0.36± 0.65	***
Security (Seg)	6.72 ± 0.54	6.90 ± 0.32	-0.18 ± 0.53	***
Tangible resources (Rt)	6.72 ± 0.47	6.89 ± 0.36	-0.17 ± 0.48	***
Response capacity (Cr)	6.76 ± 0.51	6.90 ± 0.35	-0.14± 0.53	***
Empathy (Emp)	6.79 ± 0.47	6.90± 0.31	-0.11 ± 0.46	***
Total quality	6.69 ± 0.45	6.89 ± 0.28	-0.20 ± 0.43	***

On the other hand, when considering the general quality criteria, it stands out that the interviewees expressed satisfaction and high satisfaction in relation to waiting times. Both men (35+57, n = 108) and women (125+248, n = 413) indicated feeling satisfied and very satisfied, reaching percentages of 85.19% and 90.31%, respectively. Similarly, regarding the procedures to be attended to, both men (33+58, n = 108) and women (111+277, n = 413) expressed satisfaction and high satisfaction, obtaining percentages of 84.25% and 93.94%, respectively. In terms of the general quality of health services in primary care, both men (26+71, n = 108) and women (53+336, n = 413) indicated feeling satisfied and very satisfied, reaching high percentages of 89. 81% and 94.19%, respectively. These general perceptions of satisfaction confirm the positive appreciation of the services provided in the field of Primary Health Care (PHC) by the users interviewed.

Finally, as evidenced in the concluding section of Table 2, the disparities between patients’ expectations and perceptions are statistically significant (p). The mean and standard deviation of expectations in relation to the comprehensive quality of Primary Health Care (PHC) were 6.89 ± 0.28, while the total values of perceptions reached 6.69 ± 0.45. In summary, a total gap in service quality in the APS was found to be -0.20 (± 0.43). Consequently, a significant negative gap (p) is identified, indicative of a discrepancy between the patient’s expectations and the service received (perceptions) both globally and in each of the dimensions of the PHC service.

In line with the above, the Wilcoxon test (p value W) shows that, in all cases, the deviations or gaps between patients’ expectations and perceptions are statistically significant (p). The mean and standard deviation of the expectations for the total quality of the PHC remained at 6.89 ± 0.28, and the perceptions at 6.69 ± 0.45. Finally, a total gap was recorded in the quality of PHC, which reached a differential index equal to -0.20 (p). Consequently, a significant negative gap is observed that reflects a discrepancy between the patient’s expectations and the service received, both globally and in each of the dimensions of service quality analyzed using the Servqual construct in this specific study of the APS.

## 4. Discussion

This study was designed to evaluate the quality of care in primary health services, similar to studies of the exact nature carried out in other realities, in this case by measuring the gaps between perceptions and expectations of patients [5, 44], intending to identify successes and challenges in general health services and those with less complex resolution that the PHC level of benefits delivery [10, 11]. Regarding the difficulties, deficiencies are observed in the reliability dimension (Fia), coinciding with the study carried out by Papanikolaou and Zygiaris [1] in the same context of PHC and others at the hospital level [22], which can be explained by the inability that affects the services to hire and retain high-quality professionals [19, 45]. Additionally, regarding the relative difficulty, on the one hand, to evaluate them and reward their performance and, on the other hand, to achieve sufficient medical professionals who provide quality care and communicate promptly with users [17]. Added to these difficulties is the inability to provide adequate training opportunities or spaces for the improvement needs necessary to achieve higher-quality services [10]. When it comes to analyzing the findings related to this dimension, there are coincidences with various studies [23] where it is explained that the deficiency in reliability can translate into health services delivered without the desired transparency and without counting with the required quality criteria, such as the one that is usually expected if the services are provided following the zero-error criteria postulated by the total quality criteria [8, 45].

Furthermore, a second dimension with a significant negative gap was the factor of tangible resources (Rt), referring to the availability of the necessary material means for providing services. The literature suggests that quality confronts the fact of having dense populations concerning the capacity to offer health services, in turn, presents shortages of professionals, resources, spaces, and sufficient infrastructure, which could effectively explain the significant gaps that are found. Present between expectations and perceptions [9, 13]. This fact is confirmed in the dimension of tangible resources (Rt), which in this study presented one of the highest negative gaps, confirming that physical facilities, naturally observable to users, generate perceptions below expectations [3, 34].

In PHC, dissatisfaction becomes even more evident. It is related to the observable tangible resources (Rt) that contrast with the modernity and need for updating that is required in the facilities and equipment available for the adequate provision of health services [10, 21], starting with those that are necessary for general care of a rather ambulatory nature [44] and with greater demand for those necessary for emergency and specialized health services [11]. These findings suggest the need to find an urgent solution since it is one of the reasons behind the loss of PHC-level patients who attend to receive general services in low-complexity provider centers [12, 23], all of which deteriorate the primary role of the first level of care (PHC), concentrating the demand for services by patients and family members at the most complex levels, stressing the most complex second and third level hospital services. In this context, it is known that in health and public services [13, 21], resources are limited in terms of personnel and in, time and materials. However, those available allow us to support services, reaching levels that range from the sufficient to insufficient category [3, 6], which deteriorates the perception of reliability dimensions (Fia), security (Seg) and empathy (Emp) [4, 20].

Regarding time resources, accessibility, and time pressures, members of the health personnel face stressful situations that reduce their capacities to relate with sympathy, tranquility, and appropriateness when providing ambulatory and emergency health services [18, 44]. Means and resources in practice are limited in terms of their quantity and opportunity to access to respond to vital health situations, also affecting the response capacity factor (Cr) in more specialized routine and focused services [1, 11]. Finally, the time available for attention is one of the relevant factors that affect the level of satisfaction related to the reliability dimension (Fia), which is especially relevant depending on the type, level, and resources available to provide their services [22, 45].

Regarding the sociodemographic findings, this study coincides to a great extent with Al-Momani [10] since no significant differences were reported in the quality evaluation between gender, age, and employment situation. However, they were in the level of education. However, contrary to other findings [1, 10], in this research, it was observed that those interviewed who registered higher levels of education presented lower expectations and perceptions, and even significant gaps of smaller magnitude related to previous studies and that are highly relevant when compared to studies carried out in realities as different as Nigeria and Latin America [4, 12].

Finally, even though there were significant gaps between expectation and perception in all five dimensions, as in most other reviewed studies [7, 12, 28], It is important to note that perceptions generally obtained high values [1, 21] above 6.5 points on a scale of 7, which turns out to be a highly positive finding for security services. Complementarily, the data shows that people appreciate and value PHC services, marking magnitudes whose gaps concerning expectations represent signs that must be addressed and eventually resolved, for the benefit of primary healthcare services available to citizens who access PHC-level health services, in the city of Guayaquil, Ecuador.

## 5. Conclusions

Based on the conceptual analysis and the study findings, it can be concluded that the gaps between perceptions and expectations were generally significant among women over 21 years of age, those who studied at technological and university levels, and those who work.

It is also concluded that the five dimensions of service quality of the Servqual model present negative gaps ratified with significant (p) values in all cases. Consequently, patients and family members appreciate that the expectations are not met by the perceptions of the services delivered to them in primary health care, PHC.

Among the expectations with the lowest gaps, that is, those that present some degree of satisfaction, the items of the empathy dimension are mentioned, first, acknowledging that the medical professional listened carefully and treated kindly and that, in addition, he showed interest in solving patients’ health problems. On the contrary, the reliability dimension stands out among the expectations that showed wider gaps and high degrees of dissatisfaction, also regarding the existence of hours available and ease of obtaining a medical appointment, as well as the tangible elements dimension presents gaps concerning the necessary, modern, and optimal equipment available in care rooms.

Finally, the study allows us to demonstrate the benefits of the Servqual instrument, as it collaborates effectively with the identification of the dimensions that report high degrees of satisfaction-dissatisfaction and identifies the items for which there are still unsatisfied expectations. For example, those that come from materiality factors and underlying resources, which manifested in magnitudes of negative and significant gaps that reflect discrepancies between what is expected by the patient and the service perceived at the global level of the instrument and at the level of each one of the dimensions and their respective items that must be progressively addressed by the units providing health services at the PHC level in Guayas, Ecuador.

## Supporting information

S1 Data Study database. (XLSX)

## References

[1] Papanikolaou V, Zygiaris S. Service quality perceptions in primary health care centres in Greece. Health expectations. 2014;17(2):197–207. Available from: doi: 10.1111/j.1369-7625.2011.00747.x 22296402 PMC5060715

[2] Macarayan EK, Gage AD, Doubova S V, Guanais F, Lemango ET, Ndiaye Y, et al. Assessment of quality of primary care with facility surveys: a descriptive analysis in ten low-income and middle-income countries. Lancet Glob Health. 2018;6(11):e1176–85. Available from: doi: 10.1016/S2214-109X(18)30440-6 30322648 PMC6187280

[3] Mendes IAC, Trevizan MA, de Godoy S, Nogueira PC, Ventura CAA, Furlan CEB. Expectations and perceptions of clients concerning the quality of care provided at a Brazilian hospital facility. Applied Nursing Research. 2018;39:211–6. Available from: doi: 10.1016/j.apnr.2017.11.024 29422161

[4] Boada-Niño AV, Barbosa-López AM, Cobo-Mejía EA. Perception of users regarding the quality of health care of the outpatient service according to the Servqual model. Health Research Magazine University of Boyacá. 2019; Available from: 10.24267/23897325.408

[5] Aghamolaei T, Eftekhaari TE, Rafati S, Kahnouji K, Ahangari S, Shahrzad ME, et al. Service quality assessment of a referral hospital in Southern Iran with SERVQUAL technique: patients’ perspective. BMC Health Serv Res. 2014;14(1):1–5. Available from: doi: 10.1186/1472-6963-14-322 25064475 PMC4115484

[6] Pramanik A. Patients’ perception of service quality of health care services in India: a comparative study on urban and rural hospitals. J Health Manag. 2016;18(2):205–17. Available from: http://dx.doi/10.1177/0972063416637695

[7] Teshnizi SH, Aghamolaei T, Kahnouji K, Teshnizi SMH, Ghani J. Assessing quality of health services with the SERVQUAL model in Iran. A systematic review and meta-analysis. International Journal for Quality in Health Care. 2018;30(2):82–9. Available from: doi: 10.1093/intqhc/mzx200 29408970

[8] Valencia-Arias A, Piedrahita LB, Zapata AB, Benjumea M, Moya LP. Mapping the healthcare service quality domain: A bibliometric analysis. Journal of Clinical and Diagnostic Research. 2018;12(8):IC01–5. Available from: 10.7860/JCDR/2018/30361.11863

[9] Khamis K, Njau B. Patients’ level of satisfaction on quality of health care at Mwananyamala hospital in Dar es Salaam, Tanzania. BMC Health Serv Res. 2014;14(1):1–8. Available from: doi: 10.1186/1472-6963-14-400 25230739 PMC4263110

[10] Al-Momani MM. Gap analysis between perceptions and expectations of medical-surgical patients in a public hospital in Saudi Arabia. Medical Principles and Practice. 2015;25(1):79–84. Available from: doi: 10.1159/000441000 26501371 PMC5588300

[11] Dopeykar N, Bahadori M, Mehdizadeh P, Ravangard R, Salesi M, Hosseini SM. Assessing the quality of dental services using SERVQUAL model. Dent Res J (Isfahan). 2018;15(6):430. Available from: 10.4103/1735-3327.245230 30534171 PMC6243813

[12] Adepoju OO, Opafunso Z, Ajayi M. Primary health care in south west Nigeria: Evaluating service quality and patients’ satisfaction. African Journal of Science, Technology, Innovation and Development. 2018;10(1):13–9. Available from: https://hdl.handle.net/10520/EJC-c6ff9f88b

[13] Behdioğlu S, Acar E, Burhan HA. Evaluating service quality by fuzzy SERVQUAL: a case study in a physiotherapy and rehabilitation hospital. Total Quality Management & Business Excellence. 2019;30(3–4):301–19. Available from: 10.1080/14783363.2017.1302796

[14] Aslam F, Riaz A. Impact of COVID-19 pandemic on healthcare systems: A review. J Infect Public Health. 2021;14(3):626–33.

[15] González-Carrasco I, Pérez-Santana E, González-Sánchez MA. The impact of lean six sigma on the patient satisfaction and efficiency of an emergency department. Int J Environ Res Public Health. 2019;16(2).

[16] Çelik E, Yılmaz E. Evaluation of the implementation of cloud-based quality management system in a hospital: A case study. Health Information Management Journal. 2020;49(3):116–23.

[17] Neves AS, Silva AR, Ferreira PCS. Expectation gaps versus perceptions of quality of hospital service: case study in a public hospital in Brazil. Collective Science & Health. 2019;24(12):4689–99.

[18] Gómez-Urquiza JL, Albendín-García L, Correa-Rodríguez M, Gómez-Salgado J, González-Jiménez E. Gap between expectations and perceptions of the quality of hospital service in a public hospital in Mexico. Latin American Journal of Nursery. 2019;27.

[19] Fatima I, Humayun A, Anwar MI, Iftikhar A, Aslam M, Shafiq M. How do patients perceive and expect quality of surgery, diagnostics, and emergency services in tertiary care hospitals? An evidence of gap analysis from Pakistan. Oman Med J. 2017;32(4):297. Available from: doi: 10.5001/omj.2017.58 28804582 PMC5534225

[20] Parasuraman A, Valarie AZ, Leonard LB. Servqual: A multiple-item scale for measuring consumer perc. Journal of retailing. 1988;64:12. Available from: https://www.researchgate.net/publication/225083802_SERVQUAL_A_multiple-_Item_Scale_for_measuring_consumer_perceptions_of_service_quality

[21] Purcărea VL, Gheorghe IR, Petrescu CM. The assessment of perceived service quality of public health care services in Romania using the SERVQUAL scale. Procedia Economics and Finance. 2013;6:573–85. Available from: 10.1016/S2212-5671(13)00175-5

[22] García JM, Alcaraz J. Gap between expectations and perceptions of quality of hospital service in a public hospital in Argentina. Healthcare Quality Magazine. 2018;33(3):142–7.

[23] Tripathi SN, Siddiqui MH. Assessing the quality of healthcare services: A SERVQUAL approach. Int J Healthc Manag. 2018; Available from: 10.1080/20479700.2018.1469212

[24] Júnior LDS, Rodrigues RA, Moreira DA, Maia AA. Quality of hospital services: A study in a Brazilian university hospital. Collective Science & Health. 2020;25(10):3821–40.

[25] Bustamante U.MA, Zerda E, Obando F Tello S. From Expectations to the Perception of Quality of Health Services in Guayas, Ecuador. Technological information. 2020; Available from: http://dx.doi/10.4067/S0718-07642020000100161

[26] Zapata-Copete JA, Valencia-Arias A, Palacio-Acosta CA. Quality of healthcare services in Colombia: A comparative study between public and private hospitals. BMC Health Serv Res. 2021;21(1):1–9.33388053

[27] Moreno-García MDL, Pimentel-Aguilar MA, Romero-Palacios A,. Analysis of the quality of service in a public hospital in Mexico: Patient complaints and perceptions. J Healthc Qual Res. 2019;34(1):10–8.

[28] hua Fan L, Gao L, Liu X, hong Zhao S, Mu H tong, Li Z, et al. Patients’ perceptions of service quality in China: An investigation using the SERVQUAL model. PLoS One. 2017;12(12):e0190123. Available from: doi: 10.1371/journal.pone.0190123 29272312 PMC5741236

[29] Naik Jandavath RK, Byram A. Healthcare service quality effect on patient satisfaction and behavioural intentions in corporate hospitals in India. Int J Pharm Healthc Mark. 2016 [cited 2024 Jan 27];10(1):48–74. Available from: 10.1108/IJPHM-07-2014-0043

[30] Lloret-Segura S, Ferreres-Traver A, Hernández-Baeza A, Tomás-Marco I. Exploratory factor analysis of items: a practical guide, revised and updated. Anales de psicología/annals of psychology. 2014;30(3):1151–69. Available from: 10.6018/analesps.30.3.199361.

[31] Sampieri RH, Mendoza C. Investigation methodology. Quantitative, qualitative and mixed routes. McGraw Hill México; 2018.

[32] Tang H. Qualitative Methods and Mixed Methods. Engineering Research. 2021; Available from: 10.1002/9781119624547.ch6

[33] Juárez F, López E, Villatoro J. Estadística Inferencial Univariada. Apuntes para la investigación en salud. 2014;161–282.

[34] Greenslade JH, Jimmieson NL. Organizational factors impacting on patient satisfaction: A cross sectional examination of service climate and linkages to nurses’ effort and performance. Int J Nurs Stud. 2011;48(10):1188–98. Available from: doi: 10.1016/j.ijnurstu.2011.04.004 21592476

[35] Freiberg Hoffmann A, Stover JB, de la Iglesia G, Fernández Liporace M. Polychoric and tetrachoric correlations in exploratory and confirmatory factorial studies. Ciencias Psicológicas. 2013;7(2):151–64. Available from: http://www.scielo.edu.uy/scielo.php?pid=S1688-42212013000200005&script=sci_abstract&tlng=en

[36] Pruzan P. Research methodology: the aims, practices and ethics of science. Springer; 2016.

[37] Shankar S, Singh R. Demystifying statistics: How to choose a statistical test? Indian J Rheumatol. 2014;9(2):77–81. Available from: 10.1016/j.injr.2014.04.002

[38] Makowski D, Ben-Shachar MS, Lüdecke D. bayestestR: Describing effects and their uncertainty, existence and significance within the Bayesian framework. J Open Source Softw. 2019;4(40):1541. Available from: 10.21105/joss.01541

[39] Wilcoxon F. Individual comparisons by ranking methods. In: Breakthroughs in Statistics: Methodology and Distribution. Springer; 1992. p. 196–202. Available from: 10.2307/3001968, 1(6), 80–83.

[40] Gibbons JD, Chakraborti S. Nonparametric statistical inference: revised and expanded. CRC press; 2011. Available from: 10.1007/978-3-642-04898-2_420

[41] UN WOMEN. Recognize, redistribute and reduce care work. Inspiring practices in Latin America and the Caribbean. 2018; Available from: https://lac.unwomen.org/es/digiteca/publicaciones/2018/11/estudio-reconocer-redistribuir-y-reducir-el-trabajo-de-cuidados

[42] Batthyány K, Genta N, Perrotta V. The contribution of families and women to unpaid health care in Uruguay. Feminist Studies Magazine. 2017;25:187–213. Available from: https://www.cepal.org/es/publicaciones/38911-aporte-familias-mujeres-al-cuidado-remunerado-la-salud-uruguay

[43] Sierra Leguía L, Montoya Juárez R, García Caro MP, López Morales M, Montalvo Prieto A. Family Caregiver’s Experience with Palliative and End-of-Life Care. Nursing Index. 2019;28(1–2):51–5. Available from: http://scielo.isciii.es/scielo.php?script=sci_arttext&pid=S1132-12962019000100011&lng=es&tlng=es.

[44] Cobo-Mejía EA, Gómez-Martínez FE, Rodríguez-Leal MY. Perception of the quality of care in an emergency department. Health Research Magazine University of Boyacá. 2017; Available from: 10.24267/23897325.262

[45] Vizcaíno A de J, Vizcaíno Marín V del P, Fregoso Jasso GS. Analysis of patients’ satisfaction with the emergency room services of a public hospital in Jalisco. Health Horiz. 2018;18(1):27–36. Available from: 10.19136/hs.a18n1.2103

